# Poly[[octaaqua-μ_4_-(benzene-1,2,4,5-tetra­carboxyl­ato)-dicobalt(II)] octa­hydrate]

**DOI:** 10.1107/S1600536813031577

**Published:** 2013-11-23

**Authors:** Magatte Camara, Modou Tine, Carole Daiguebonne, Olivier Guillou, Thierry Roisnel

**Affiliations:** a Université Assane Seck de Ziguinchor, LCPM, Groupe Matériaux Inorganiques, Chimie Douce et Cristallographie, BP 523 Ziguinchor, Senegal; b INSA, UMR 6226, Institut des Sciences Chimiques de Rennes, 35708 Rennes, France; c Institut des Sciences Chimiques de Rennes, UMR CNRS 6226, Université de Rennes I, Avenue du Général Leclerc, 35042 Rennes Cedex, France

## Abstract

The title polymeric coordination compound, {[Co_2_(C_10_H_2_O_8_)(H_2_O)_8_]·8H_2_O}_*n*_, was obtained by slow diffusion of a dilute aqueous solution of CoCl_2_ and the sodium salt of benzene-1,2,4,5-tetracarboxylic acid (H_4_btec) through an agar–agar gel bridge in a U-shaped tube. The two independent Co^2+^ ions are each situated on an inversion centre and are coordinated in a slightly distorted octa­hedral geometry by four water O atoms and two carboxyl­ate O atoms from two btec^4−^ ligands (-1> symmetry), forming a layer parallel to (11-1). This layer can be described as a mol­ecular two-dimensional square grid with the benzene rings at the nodes and the Co^II^ atoms connecting the nodes. O—H⋯O hydrogen-bonding interactions involving the coordinating water molecules, the carboxylate O atoms and lattice water molecules lead to the formation of a three-dimensional network.

## Related literature

For related metal-organic materials with large channels and cavities, see: Yaghi *et al.* (1998 ▶); Evans *et al.* (1999 ▶); Eddaoudi *et al.* (2002 ▶); Guillou *et al.* (2006 ▶). For examples of coordination polymers containing the btec^4−^ ligand, see: Cheng *et al.* (2000 ▶); Rochon & Massarweh (2000 ▶); Chu *et al.* (2001 ▶); Wu *et al.* (2002 ▶); Luo *et al.* (2013 ▶). For related crystal-growth methods in gels, see: Henisch & Rustum (1970 ▶); Henisch (1988 ▶); Daiguebonne *et al.* (2003 ▶). 
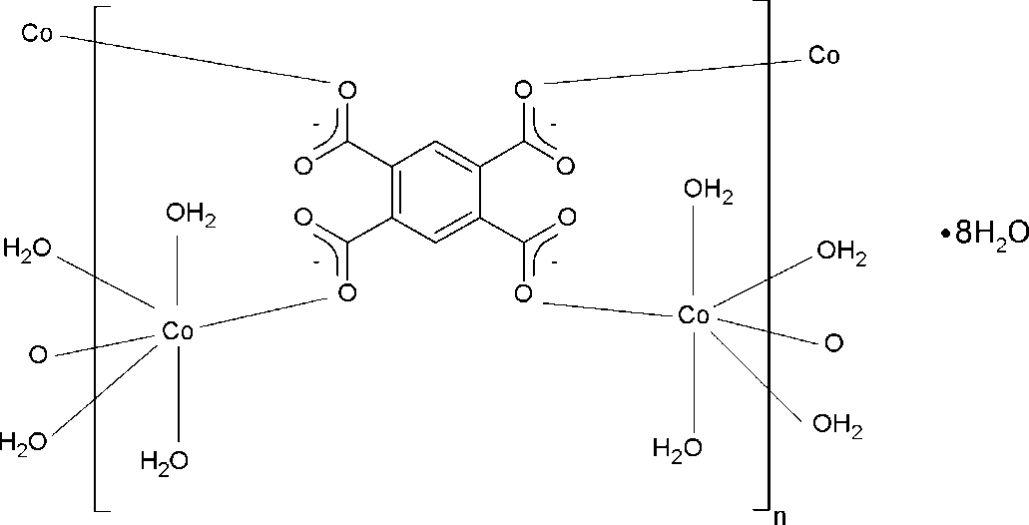


## Experimental

### 

#### Crystal data

[Co_2_(C_10_H_2_O_8_)(H_2_O)_8_]·8H_2_O
*M* *_r_* = 656.22
Triclinic, 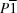

*a* = 5.4371 (1) Å
*b* = 9.8496 (3) Å
*c* = 10.2564 (3) Å
α = 96.445 (1)°
β = 91.232 (1)°
γ = 91.328 (1)°
*V* = 545.48 (3) Å^3^
*Z* = 1
Mo *K*α radiation
μ = 1.64 mm^−1^
*T* = 298 K
0.10 × 0.09 × 0.06 mm

#### Data collection

Bruker APEXII diffractometer
8487 measured reflections
2456 independent reflections
2092 reflections with *I* > 2σ(*I*)
*R*_int_ = 0.025

#### Refinement

*R*[*F* ^2^ > 2σ(*F* ^2^)] = 0.048
*wR*(*F* ^2^) = 0.153
*S* = 0.97
2456 reflections
166 parameters
H-atom parameters constrained
Δρ_max_ = 0.60 e Å^−3^
Δρ_min_ = −0.81 e Å^−3^

### 

Data collection: *APEX2* (Bruker, 2007 ▶); cell refinement: *SAINT* (Bruker, 2007 ▶); data reduction: *SAINT*; program(s) used to solve structure: *SHELXS97* (Sheldrick, 2008 ▶); program(s) used to refine structure: *SHELXL2013* (Sheldrick, 2008 ▶); molecular graphics: *DIAMOND* (Brandenburg, 1999 ▶); software used to prepare material for publication: *publCIF* (Westrip, 2010 ▶).

## Supplementary Material

Crystal structure: contains datablock(s) I. DOI: 10.1107/S1600536813031577/vn2077sup1.cif

Structure factors: contains datablock(s) I. DOI: 10.1107/S1600536813031577/vn2077Isup2.hkl

Additional supplementary materials:  crystallographic information; 3D view; checkCIF report

## Figures and Tables

**Table 1:** Selected bond lengths (Å)

Co1—O1	2.084 (3)
Co1—O3	2.089 (3)
Co1—O23	2.106 (2)
Co2—O2	2.101 (3)
Co2—O4	2.060 (3)
Co2—O12	2.122 (2)

**Table 2:** Hydrogen-bond geometry (Å, °)

*D*—H⋯*A*	*D*—H	H⋯*A*	*D*⋯*A*	*D*—H⋯*A*
O3—H1⋯O11^i^	0.79	1.92	2.698 (4)	169
O4—H4⋯O14	0.82	1.89	2.636 (9)	150
O4—H4⋯O16	0.82	1.92	2.632 (10)	143
O1—H5⋯O23^ii^	0.89	1.87	2.746 (4)	171
O2—H6⋯O12^iii^	0.89	1.92	2.803 (4)	177
O1—H7⋯O22^iv^	0.75	1.97	2.664 (4)	154
O2—H9⋯O22	0.90	1.82	2.725 (4)	176
O3—H10⋯O14	0.73	1.95	2.682 (9)	176
O3—H10⋯O15^v^	0.73	2.32	2.848 (9)	130
O4—H13⋯O11^vi^	0.73	1.94	2.618 (5)	154

Symmetry codes: (i) 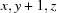; (ii) 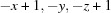; (iii) 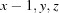; (iv) 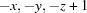; (v) 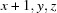; (vi) 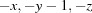.

**Fig. 1.**
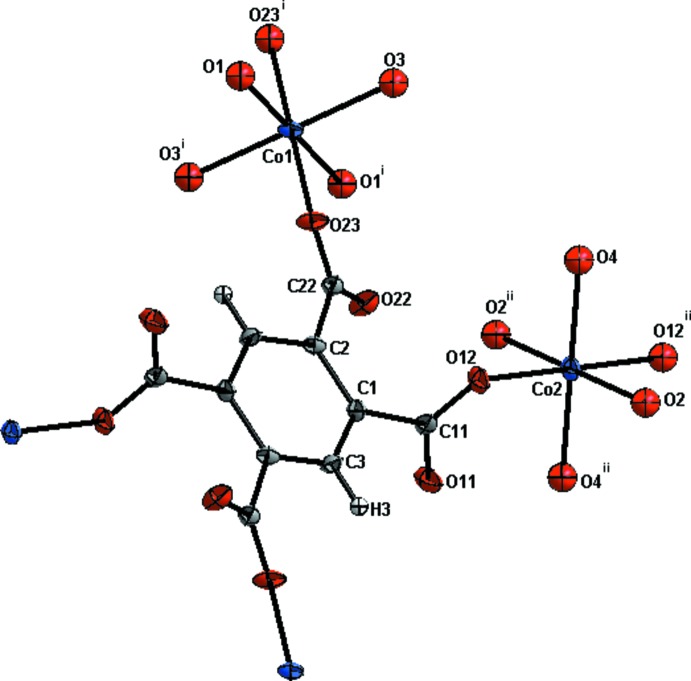
Extended asymetric unit of the title compound, with the atom-numbering scheme. Displacement ellipsoids are drawn at the 50% probability level. [Symmetry codes: (i) -x, -y, -z + 1; (ii) -x, -y - 1, -z; (iii) -x + 1, -y - 1, -z + 1.]

**Fig. 2.**
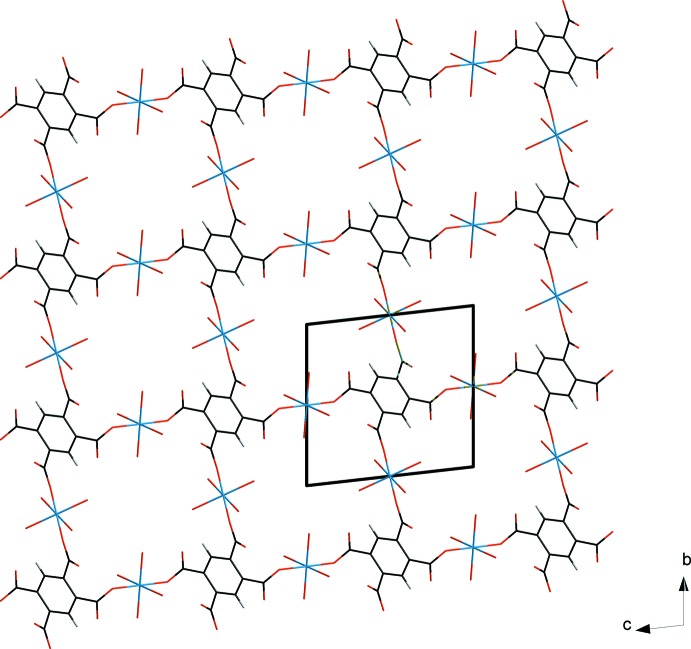
View along the a axis of the molecular square grid layer of the title compound.

## supplementary crystallographic information

### 1. Comment

The design of metal-organic materials with large channels and cavities has been deeply investigated, due to their intriguing structural diversity and potential functions as microporous solids for molecular adsorption, ion exchange, and heterogeneous catalysis. For related structures in this context, see: Yaghi *et al.*, 1998; Evans *et al.*, 1999; Eddaoudi *et al.*, 2002; Guillou *et al.*, 2006. The benzene-1,2,4,5-tetracarboxylate ligand (btec^4-^) as a multi-connecting ligand is an excellent candidate for the design of coordination polymers. Surprisingly, examples of coordination polymers involving this ligand are relatively scarce. For examples of coordination polymers with this ligand, see: Cheng *et al.*, 2000; Chu *et al.*, 2001; Rochon & Massarweh, 2000; Wu *et al.*, 2002; Luo *et al.*, 2013). We report here the synthesis and the crystal structure of the title coordination polymer.

All carboxylic groups of the organic ligand in the title compound are deprotonated and each of them adopts a monodentate coordination mode. There are two crystallographically independent Co^II^ atoms in the structure. In both cases the Co^II^ atoms are coordinated by two carboxylate oxygen atoms from two btec ligands and four water oxygen atoms (Fig. 1). There is no significant difference in the coordination distance between carboxyl and water oxygen atoms. The coordination Co···O distances in the title polymeric compound range from 2.067 (3) to 2.129 (3) Å. Each btec^4-^ ligand links four Co^II^ atoms and each Co^II^ atom is bond to two btec^4-^ ligands to form a two-dimensional layer. These layers can be described as a molecular two-dimensional square grid in which the phenyl rings are at the nodes and the Co^II^ atoms connecting the nodes (Fig. 2). The area of the square cells of the grids is larger than 120 Å^2^ (11.3 Å *x* 11.5 Å). The crystal packing is enforced by a complex hydrogen bonds network that involves the crystallization water molecules located in the inter-layer space.

### 2. Experimental

All reagents were used as obtained without further purification. Cobalt chloride was purchased from STREM Chemicals. benzene-1,2,4,5-tetracarboxylic acid was purchased from Acros Organics. Its sodium salt was prepared by addition of four equivalents of sodium hydroxide to a suspension of benzene-1,2,4,5-tetracaboxylic acid in de-ionized water until complete dissolution. Then, the solution was evaporated to dryness. The solid phase was then put in suspension in ethanol, stirred and refluxed during 1 h. After filtration and drying in a desiccator, a white powder of tetra-sodium benzene-1,2,4,5-tetracarboxylate was obtained. The yield of this synthesis is 90%.

Single crystals of the coordination polymer were obtained by slow diffusion of dilute aqueous solutions of Co(II) chloride (0.25 mmol in 20 ml) and of sodium salt of benzene-1,2,4,5-tetracarboxylic acid (0.25 mmol in 20 mL) through an agar-agar gel bridge in a U-shaped tube. The gel was purchased from Acros Organics and jellified according to established procedure. For a related procedure, see: Henisch & Rustum, 1970; Henisch, 1988; Daiguebonne *et al.*, 2003. After several weeks, very light pink single crystals were obtained.

### 3. Refinement

H-atoms from crystallization water molecules could not be assigned reliably and were thus not included in the refinement, but they were taken into account for the chemical formula sum, moiety, weight, as well as for the absorption coefficient and the number of electrons in the unit cell.

### Crystal data

**Table:**

[Co_2_(C_10_H_2_O_8_)(H_2_O)_8_]·8H_2_O	*V* = 545.48 (3) Å^3^
*M**_r_* = 656.22	*Z* = 1
Triclinic, *P*1	*F*(000) = 340
*a* = 5.4371 (1) Å	*D*_x_ = 1.998 Mg m^−^^3^
*b* = 9.8496 (3) Å	Mo *K*α radiation, λ = 0.71073 Å
*c* = 10.2564 (3) Å	µ = 1.64 mm^−^^1^
α = 96.445 (1)°	*T* = 298 K
β = 91.232 (1)°	Prism, very light pink
γ = 91.328 (1)°	0.10 × 0.09 × 0.06 mm

### Data collection

**Table:**

Bruker APEXII diffractometer	2092 reflections with *I* > 2σ(*I*)
Radiation source: Fine-focus sealed tube	*R*_int_ = 0.025
Graphite monochromator	θ_max_ = 27.5°, θ_min_ = 2.0°
CCD rotation images, thin slices scans	*h* = −6→7
8487 measured reflections	*k* = −12→12
2456 independent reflections	*l* = −13→13

### Refinement

**Table:**

Refinement on *F*^2^	0 restraints
Least-squares matrix: full	Hydrogen site location: difference Fourier map
*R*[*F*^2^ > 2σ(*F*^2^)] = 0.048	H-atom parameters constrained
*wR*(*F*^2^) = 0.153	*w* = 1/[σ^2^(*F*_o_^2^) + (0.0911*P*)^2^ + 2.3818*P*] where *P* = (*F*_o_^2^ + 2*F*_c_^2^)/3
*S* = 0.97	(Δ/σ)_max_ < 0.001
2456 reflections	Δρ_max_ = 0.60 e Å^−^^3^
166 parameters	Δρ_min_ = −0.81 e Å^−^^3^

### Special details

**Table:**

Geometry. All e.s.d.'s (except the e.s.d. in the dihedral angle between two l.s. planes) are estimated using the full covariance matrix. The cell e.s.d.'s are taken into account individually in the estimation of e.s.d.'s in distances, angles and torsion angles; correlations between e.s.d.'s in cell parameters are only used when they are defined by crystal symmetry. An approximate (isotropic) treatment of cell e.s.d.'s is used for estimating e.s.d.'s involving l.s. planes.

### Fractional atomic coordinates and isotropic or equivalent isotropic displacement parameters (Å^2^)

**Table:**

	*x*	*y*	*z*	*U*_iso_*/*U*_eq_	
H1	0.0869	0.1434	0.3071	0.050*	
H13	0.0780	−0.2852	−0.0807	0.050*	
H4	0.2169	−0.2460	0.0208	0.050*	
H5	0.4549	0.1226	0.5668	0.050*	
H6	−0.4194	−0.4465	0.1454	0.050*	
H7	0.2609	0.1784	0.6194	0.050*	
H9	−0.1925	−0.3972	0.2127	0.050*	
H10	0.1381	0.0269	0.2641	0.050*	
Co1	0.0000	0.0000	0.5000	0.0165 (2)	
Co2	0.0000	−0.5000	0.0000	0.0180 (2)	
O22	−0.0419 (5)	−0.3362 (3)	0.3694 (3)	0.0246 (6)	
O12	0.2439 (5)	−0.5016 (3)	0.1641 (3)	0.0212 (6)	
O23	0.2189 (5)	−0.1729 (3)	0.4617 (3)	0.0224 (6)	
O11	0.0733 (6)	−0.6758 (3)	0.2541 (3)	0.0352 (8)	
O1	0.3013 (5)	0.1098 (3)	0.5906 (3)	0.0308 (7)	
O2	−0.2644 (6)	−0.4222 (4)	0.1330 (3)	0.0399 (8)	
C1	0.3561 (6)	−0.5310 (4)	0.3841 (3)	0.0159 (7)	
O3	0.0735 (7)	0.0657 (3)	0.3177 (3)	0.0381 (8)	
C3	0.5172 (7)	−0.6259 (4)	0.4275 (3)	0.0170 (7)	
H3	0.5283	−0.7109	0.3786	0.020*	
C2	0.3387 (6)	−0.4025 (3)	0.4587 (3)	0.0150 (7)	
C22	0.1578 (6)	−0.2968 (4)	0.4251 (3)	0.0158 (7)	
C11	0.2111 (7)	−0.5719 (4)	0.2586 (3)	0.0180 (7)	
O4	0.1143 (9)	−0.3016 (4)	−0.0152 (4)	0.0596 (13)	
O14	0.2936 (16)	−0.0732 (8)	0.1144 (8)	0.121 (3)	
O16	0.5551 (16)	−0.1850 (8)	0.0171 (9)	0.121 (3)	
O15	−0.4581 (14)	0.0456 (9)	0.1989 (8)	0.116 (2)	
O17	0.8066 (16)	−0.0714 (9)	0.1011 (8)	0.124 (3)	

### Atomic displacement parameters (Å^2^)

**Table:**

	*U*^11^	*U*^22^	*U*^33^	*U*^12^	*U*^13^	*U*^23^
Co1	0.0162 (4)	0.0120 (3)	0.0218 (4)	0.0031 (2)	−0.0002 (3)	0.0034 (3)
Co2	0.0196 (4)	0.0198 (4)	0.0146 (3)	−0.0009 (3)	−0.0048 (2)	0.0036 (3)
O22	0.0202 (13)	0.0222 (13)	0.0296 (14)	0.0064 (10)	−0.0078 (11)	−0.0047 (11)
O12	0.0217 (13)	0.0263 (14)	0.0160 (12)	−0.0007 (11)	−0.0063 (10)	0.0054 (10)
O23	0.0186 (13)	0.0124 (12)	0.0362 (15)	0.0043 (10)	−0.0029 (11)	0.0023 (11)
O11	0.051 (2)	0.0243 (15)	0.0307 (16)	−0.0129 (14)	−0.0227 (14)	0.0108 (12)
O1	0.0192 (14)	0.0204 (14)	0.0511 (19)	0.0020 (11)	−0.0027 (12)	−0.0030 (13)
O2	0.0224 (15)	0.073 (3)	0.0221 (15)	0.0016 (15)	−0.0025 (11)	−0.0034 (15)
C1	0.0176 (16)	0.0160 (16)	0.0140 (15)	0.0013 (13)	−0.0038 (12)	0.0022 (13)
O3	0.064 (2)	0.0211 (15)	0.0307 (16)	0.0035 (14)	0.0150 (15)	0.0072 (12)
C3	0.0207 (17)	0.0121 (15)	0.0175 (16)	0.0027 (13)	−0.0029 (13)	−0.0011 (12)
C2	0.0161 (16)	0.0131 (15)	0.0161 (16)	0.0032 (12)	−0.0009 (12)	0.0027 (12)
C22	0.0155 (16)	0.0162 (16)	0.0157 (16)	0.0032 (13)	0.0000 (12)	0.0014 (13)
C11	0.0217 (17)	0.0147 (16)	0.0173 (17)	0.0063 (13)	−0.0062 (13)	0.0003 (13)
O4	0.099 (3)	0.045 (2)	0.0354 (19)	−0.040 (2)	−0.038 (2)	0.0205 (16)
O14	0.141 (7)	0.106 (6)	0.112 (6)	−0.001 (5)	0.012 (5)	−0.009 (4)
O16	0.127 (6)	0.106 (5)	0.131 (6)	−0.003 (5)	−0.023 (5)	0.025 (5)
O15	0.105 (5)	0.146 (7)	0.101 (5)	0.022 (5)	−0.002 (4)	0.022 (5)
O17	0.142 (7)	0.127 (6)	0.101 (5)	0.020 (5)	−0.017 (5)	0.004 (5)

### Geometric parameters (Å, º)

**Table:**

Co1—O1	2.084 (3)	Co2—O12	2.122 (2)
Co1—O1^i^	2.084 (3)	O22—C22	1.249 (4)
Co1—O3	2.089 (3)	O12—C11	1.266 (5)
Co1—O3^i^	2.089 (3)	O23—C22	1.270 (4)
Co1—O23	2.106 (2)	O11—C11	1.250 (5)
Co1—O23^i^	2.106 (2)	C1—C3	1.398 (5)
Co2—O2	2.101 (3)	C1—C2	1.410 (5)
Co2—O4	2.060 (3)	C1—C11	1.505 (5)
Co2—O4^ii^	2.061 (3)	C3—C2^iii^	1.390 (5)
Co2—O2^ii^	2.101 (3)	C2—C3^iii^	1.390 (5)
Co2—O12^ii^	2.122 (2)	C2—C22	1.511 (5)
			
O1—Co1—O1^i^	180.0	O2^ii^—Co2—O12^ii^	87.12 (11)
O1—Co1—O3	92.62 (14)	O2—Co2—O12^ii^	92.88 (11)
O1^i^—Co1—O3	87.38 (14)	O4—Co2—O12	88.91 (12)
O1—Co1—O3^i^	87.38 (14)	O4^ii^—Co2—O12	91.09 (12)
O1^i^—Co1—O3^i^	92.62 (14)	O2^ii^—Co2—O12	92.88 (11)
O3—Co1—O3^i^	180.0	O2—Co2—O12	87.12 (11)
O1—Co1—O23	90.10 (11)	O12^ii^—Co2—O12	180.0
O1^i^—Co1—O23	89.90 (11)	C11—O12—Co2	124.9 (2)
O3—Co1—O23	92.24 (12)	C22—O23—Co1	130.4 (2)
O3^i^—Co1—O23	87.76 (12)	C3—C1—C2	118.8 (3)
O1—Co1—O23^i^	89.90 (11)	C3—C1—C11	117.7 (3)
O1^i^—Co1—O23^i^	90.10 (11)	C2—C1—C11	123.5 (3)
O3—Co1—O23^i^	87.76 (12)	C2^iii^—C3—C1	122.2 (3)
O3^i^—Co1—O23^i^	92.24 (12)	C3^iii^—C2—C1	119.0 (3)
O23—Co1—O23^i^	180.0	C3^iii^—C2—C22	118.1 (3)
O4—Co2—O4^ii^	180.0	C1—C2—C22	122.8 (3)
O4—Co2—O2^ii^	91.81 (19)	O22—C22—O23	124.9 (3)
O4^ii^—Co2—O2^ii^	88.19 (19)	O22—C22—C2	118.8 (3)
O4—Co2—O2	88.19 (19)	O23—C22—C2	116.2 (3)
O4^ii^—Co2—O2	91.81 (19)	O11—C11—O12	124.9 (3)
O2^ii^—Co2—O2	180.0	O11—C11—C1	117.4 (3)
O4—Co2—O12^ii^	91.09 (12)	O12—C11—C1	117.7 (3)
O4^ii^—Co2—O12^ii^	88.91 (12)		

Symmetry codes: (i) −*x*, −*y*, −*z*+1; (ii) −*x*, −*y*−1, −*z*; (iii) −*x*+1, −*y*−1, −*z*+1.

### Hydrogen-bond geometry (Å, º)

**Table:**

*D*—H···*A*	*D*—H	H···*A*	*D*···*A*	*D*—H···*A*
O3—H1···O11^iv^	0.79	1.92	2.698 (4)	169
O4—H4···O14	0.82	1.89	2.636 (9)	150
O4—H4···O16	0.82	1.92	2.632 (10)	143
O1—H5···O23^v^	0.89	1.87	2.746 (4)	171
O2—H6···O12^vi^	0.89	1.92	2.803 (4)	177
O1—H7···O22^i^	0.75	1.97	2.664 (4)	154
O2—H9···O22	0.90	1.82	2.725 (4)	176
O3—H10···O14	0.73	1.95	2.682 (9)	176
O3—H10···O15^vii^	0.73	2.32	2.848 (9)	130
O4—H13···O11^ii^	0.73	1.94	2.618 (5)	154

Symmetry codes: (i) −*x*, −*y*, −*z*+1; (ii) −*x*, −*y*−1, −*z*; (iv) *x*, *y*+1, *z*; (v) −*x*+1, −*y*, −*z*+1; (vi) *x*−1, *y*, *z*; (vii) *x*+1, *y*, *z*.
